# Generating viral metagenomes from the coral holobiont

**DOI:** 10.3389/fmicb.2014.00206

**Published:** 2014-05-07

**Authors:** Karen D. Weynberg, Elisha M. Wood-Charlson, Curtis A. Suttle, Madeleine J. H. van Oppen

**Affiliations:** ^1^Australian Institute of Marine ScienceTownsville, QLD, Australia; ^2^Department of Microbiology and Immunology, University of British ColumbiaVancouver, BC, Canada; ^3^Department of Earth, Ocean and Atmospheric Sciences, University of British ColumbiaVancouver, BC, Canada; ^4^Department of Botany, University of British ColumbiaVancouver, BC, Canada; ^5^Canadian Institute for Advanced Research, University of British ColumbiaVancouver, BC, Canada

**Keywords:** viral metagenomics, coral, holobiont, chloroform, virus diversity

## Abstract

Reef-building corals comprise multipartite symbioses where the cnidarian animal is host to an array of eukaryotic and prokaryotic organisms, and the viruses that infect them. These viruses are critical elements of the coral holobiont, serving not only as agents of mortality, but also as potential vectors for lateral gene flow, and as elements encoding a variety of auxiliary metabolic functions. Consequently, understanding the functioning and health of the coral holobiont requires detailed knowledge of the associated viral assemblage and its function. Currently, the most tractable way of uncovering viral diversity and function is through metagenomic approaches, which is inherently difficult in corals because of the complex holobiont community, an extracellular mucus layer that all corals secrete, and the variety of sizes and structures of nucleic acids found in viruses. Here we present the first protocol for isolating, purifying and amplifying viral nucleic acids from corals based on mechanical disruption of cells. This method produces at least 50% higher yields of viral nucleic acids, has very low levels of cellular sequence contamination and captures wider viral diversity than previously used chemical-based extraction methods. We demonstrate that our mechanical-based method profiles a greater diversity of DNA and RNA genomes, including virus groups such as Retro-transcribing and ssRNA viruses, which are absent from metagenomes generated via chemical-based methods. In addition, we briefly present (and make publically available) the first paired DNA and RNA viral metagenomes from the coral *Acropora tenuis*.

## Introduction

Marine viruses are the most abundant biological agents in the world's oceans, with quantities exceeding bacterial abundances by at least one order of magnitude (Wommack and Colwell, 2000; Suttle, 2005, 2007), and can infect members of all domains of life (Rohwer et al., 2009). Viruses play fundamental roles in the evolution and population dynamics of their hosts and are key players in oceanic biogeochemical cycling (Suttle, 2005). Despite more than two decades of research into viruses in the marine environment, comparatively little research has been conducted on viruses associated with coral reefs. The term “coral holobiont” describes the entirety of the community comprised by a coral colony and includes the coral animal, as well as microscopic organisms including symbiotic dinoflagellates and endolithic algae, bacteria, fungi, archaea, and viruses (Rohwer et al., 2002). Over the past decade a range of methods have been employed to study viruses associated with coral reefs and the coral holobiont, including transmission electron microscopy (TEM) (Wilson et al., 2001, 2005a; Davy et al., 2006; Davy and Patten, 2007; Lohr et al., 2007; Patten et al., 2008a), flow cytometry (Seymour et al., 2005; Patten et al., 2006, 2008b; Lohr et al., 2007), and metagenomics (Angly et al., 2006; Dinsdale et al., 2008; Marhaver et al., 2008; Thurber et al., 2008; Hewson et al., 2012). While these studies have revealed a diverse array of viruses, coral virology is in its infancy and inherent methodological challenges still exist in this research field. Currently, there are no established coral cell lines and only two bacteriophages have been isolated and characterized; they infect the coral-associated bacterial pathogens, *Vibrio coralliilyticus* (Efrony et al., 2007) and *Thallasomonas loyana* (Efrony et al., 2009).

Viral metagenomics is defined here as the study of viral genetic material contained within an environmental sample. Metagenomics is a relatively new and promising tool for characterizing coral-associated viral communities but it also faces certain pitfalls and limitations that can influence data interpretation. Caveats include the limited number of viral sequences currently in public databases, biases introduced during the isolation of viral metagenomes from coral tissues and the complexity of the multi-compartmental nature of the coral holobiont.

The main experimental objective of this study was to develop a method that best captures the diversity of both DNA and RNA viruses associated with the coral holobiont as assessed by metagenomics. We compared our new method with existing methods for isolating viral metagenomes from corals (Marhaver et al., 2008; Thurber et al., 2008, 2009; Hewson et al., 2012). These methods notably include a chloroform addition step, prior to virus purification, and we demonstrate this can lead to exclusion of certain virus families in the final data. We also show that the amplification methods used can influence the data arising from sequencing viral metagenomes in coral tissue. Our method reduces potential biases for certain viral groups and captures a larger diversity of the viral community in coral tissues compared to other methods (Marhaver et al., 2008; Thurber et al., 2008, 2009; Hewson et al., 2012). We initially validated this method for isolating viral DNA from the coral, *Pocillopora damicornis* (Pocilloporidae), and subsequently for isolating viral DNA and RNA from a member of the Acroporidae (*Acropora tenuis*). Both families are ecologically important in Indo-Pacific coral reef systems (Veron, 1993).

## Materials and methods

### Sampling locations and collection of coral tissue

Field sampling occurred at Trunk Reef (18°20′49″S, 146°49′46″E) in November 2012 and in Pioneer Bay off Orpheus Island (18°38′3″S, 146°29′57″E) in March 2013, in the central Great Barrier Reef. At Trunk Reef, approximately 45 g of coral tissue was sampled from three healthy, freshly collected coral colonies of *Pocillopora damicornis*. Approximately 20 g of *Acropora tenuis* tissue was sampled from three healthy, freshly collected coral colonies collected in Pioneer Bay. Fragments were washed in autoclaved, 0.02 μm filtered virus-free seawater. Subsequently, tissue was blasted from the coral skeleton, using an air-gun, into 15 mL 0.02 μm filtered (Anotop, Whatman) SM buffer (100 mM NaCl, 8 mM MgSO_4_, 50 mM Tris pH 7.5) in a zip-lock bag.

### Chloroform extraction and cesium chloride density gradient centrifugation

For the *P. damicornis* samples, isolation of the viral metagenomes associated with the coral tissue was undertaken in a 3-way comparison of methodologies. The first approach was to replicate previously published protocols for isolating viruses from coral tissue (Marhaver et al., 2008; Thurber et al., 2008, 2009) using a chloroform disruption step, which we term the chloroform (CFM) method. Briefly, 5 mL of chloroform per 40 mL of coral blastate was added and samples were agitated gently for 1 h at room temperature. Coral blastates were homogenized at 5000 rpm for 1 min (Heidolph SilentCrusher™). Samples were immediately centrifuged at 1000 g for 15 min. The supernatant was transferred to sterile glass corex tubes and spun at 12,000 g for 15 min to pellet the majority of microbial cells (Beckman Coulter JA 25.50 rotor). A cesium chloride (CsCl) density gradient was then formed by layering 1 mL of 1.7, 1.5, and 1.35 g mL^−1^ CsCl into 13.2 mL UltraClear™ ultracentrifuge tubes (Beckman Coulter) with 9 mL sample layered on the top of the gradient. Gradients were then centrifuged for 2 h at 60,000 g at 4°C in a swinging bucket rotor.

### New mechanical-based method omitting the use of chloroform

**Figure 1:**
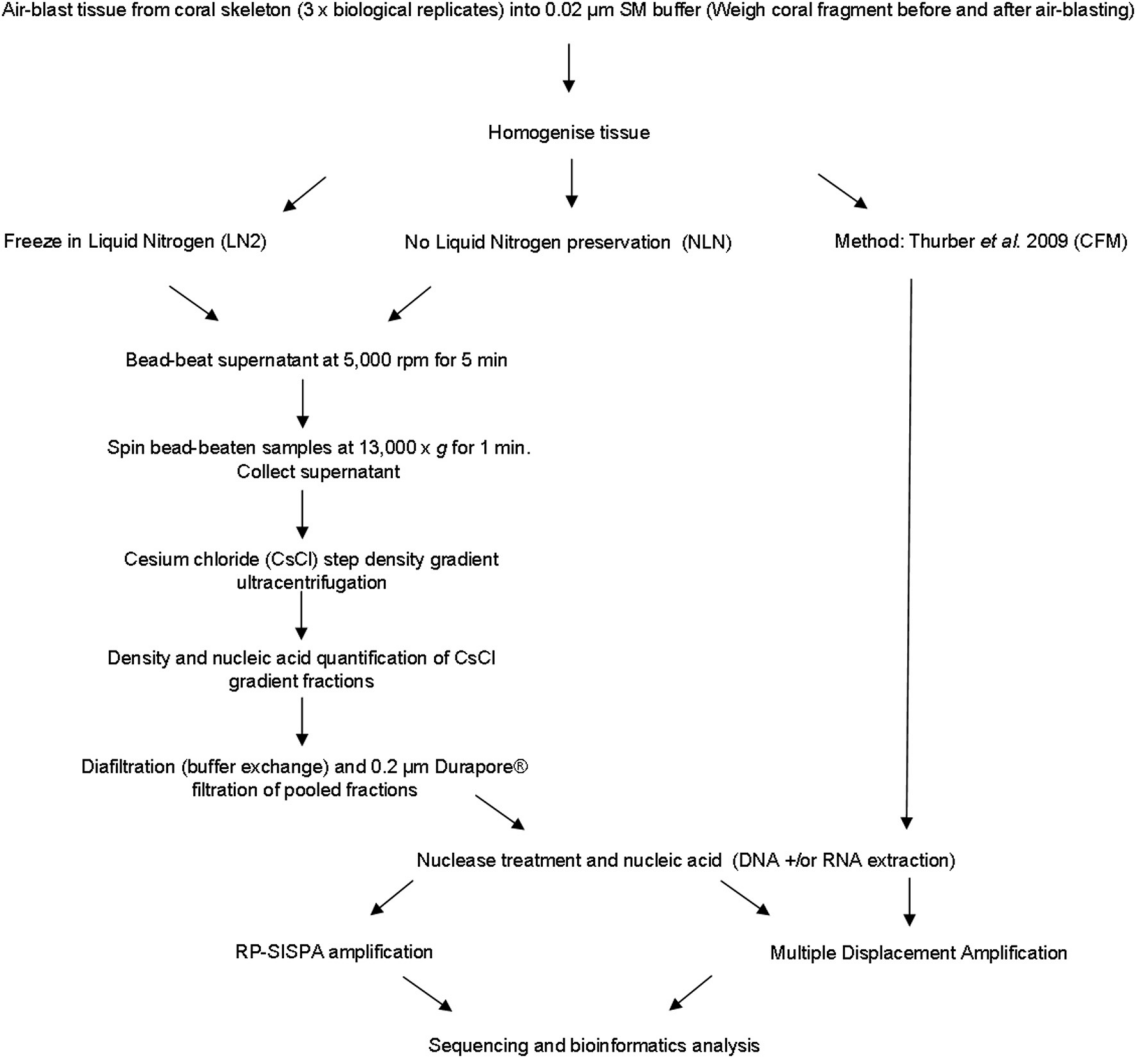
**Flowchart outlining the methods for generating viral metagenomes from coral tissue trialed in this study**.

A number of viruses are sensitive to chloroform as it acts to remove the lipid envelope surrounding the exterior of the viral capsid (Feldman and Wang, 1961; Ackermann, 2006). We developed an alternative approach to avoid the use of chloroform, instead using mechanical disruption to break open host cells, which we term the mechanical (MECH) method. In addition, the MECH protocol was used to test the effects of storage in liquid nitrogen on samples post-tissue homogenization. Sampling of coral colonies in the field frequently means working in remote locations with limited access to resources specific to virus purification, such as an ultracentrifuge. Therefore, preservation of fresh coral tissue homogenate using liquid nitrogen is often necessary until further processing in a laboratory can occur. We tested the effect of storage in liquid nitrogen (LN2) prior to nucleic-acid isolation vs. the immediate processing of fresh coral tissue homogenate (no liquid nitrogen, NLN). A simplified outline of the optimized method using MECH for generating viral metagenomes from coral tissue is shown in Figure 1. To standardize the sample for method testing, we pooled tissue homogenate from three *P. damicornis* colonies and subdivided the pooled sample for processing by MECH (NLN and LN2) and the published CFM method for viral metagenome isolation, purification and amplification.

In the MECH method, the coral tissue blastate was homogenized at 10,000 rpm for 1 min and spun at 400 g for 5 min. The supernatant was then aliquoted into 1.5 mL aliquots in 2 mL eppendorf tubes containing 0.3 mL acid-washed glass beads (425–600 μm diameter) (Sigma-Aldrich). The tubes were placed in a bead beater and cells were disrupted at 5000 rpm for 5 min. Tubes were centrifuged at top speed in a bench-top Eppendorf centrifuge for 1 min and the supernatant was collected for viral fractionation using step CsCl density gradients. To confirm that the MECH method was not disrupting virus particles, two dsDNA viruses, OtV-2 (Weynberg et al., 2011) and EhV-86 (Wilson et al., 2005b), were subjected to the same mechanical disruption protocol. Flow cytometry was used to enumerate viruses before and after disruption. We could find no discernible differences in virus populations following MECH (data not shown).

CsCl solutions were made with solid molecular-grade CsCl (Sigma-Aldrich) dissolved in 0.02 μm filtered (Anotop, Whatman) SM buffer. A 3 mL cushion of 1.6 g mL^−1^ CsCl was added to the bottom of a 13.2 mL UltraClear™ ultracentrifuge tube (Beckman Coulter). Next, 2.5 mL of 1.45 g mL^−1^and 1.3 g mL^−1^ densities and 2 mL of 1.2 g mL^−1^ were sequentially layered in that order on top of the 1.6 g mL^−1^ layer. The density of sample homogenate supernatant was adjusted to 1.12 g mL^−1^ with CsCl and 2 mL of sample was placed on top of the layered gradient. Gradients were then centrifuged in an Optima XL-80K ultracentrifuge (Beckman Coulter) in a swinging bucket rotor (SW 41 Ti, Beckman Coulter) for 2.5 h at 40,000 rpm and 4°C. Fractions (0.5 mL) from the gradients were collected in 1.5 mL tubes using an 18 bore gauge needle and luer-lok syringe, puncturing the tube ~1 mL from the bottom.

The density of fractions was determined gravimetrically and DNA concentration of each fraction was measured using a Quant-It Picogreen dsDNA High Sensitivity assay kit (Invitrogen, Life Technologies). Diafiltration and buffer exchange were performed to remove CsCl salts, as their presence may interfere with downstream processing, such as DNA extraction. Fractions containing the nucleic acid peaks were pooled and buffer exchange was performed with Amicon® centrifugal spin columns (30 kDa, Millipore) against 0.02 μm filtered SM buffer. The diafiltrated sample was then filtered using a 0.2 μm pore size Durapore® syringe filter to remove remaining contaminating bacteria.

### Nucleic acid extraction and amplification for sequencing

All samples were treated with DNase and RNase (Ambion) prior to nucleic acid extraction. DNA was extracted and RNase treated using a MasterPure kit (Epicentre, Illumina) following manufacturer's instructions. RNA was extracted using a Qiagen QIAamp viral RNA kit (Qiagen) following manufacturer's instructions, including the final DNase step (Ambion). Each amplification method is outlined in detail below. The amplification methods used were Phi 29 polymerase-based Multiple Displacement Amplification (MDA) RepliG® (QIAGEN) technology and a modified Random Priming-mediated Sequence-Independent Single-Primer Amplification (RP-SISPA) approach. Samples processed via the CFM method were only amplified using RepliG®, as this is similar to other Phi 29 polymerase-based amplification techniques used in this method (e.g., GenomiPhi®) (Thurber et al., 2008; Hewson et al., 2012). The NLN and LN2 samples were amplified using both RepliG® and a RP-SISPA method for DNA viral metagenomes modified from a published protocol for amplifying RNA viruses extracted from seawater samples (Culley et al., 2010). The modified method converts viral DNA to dsDNA through a two-step Klenow reaction, which also adds the primer sites to both DNA strands prior to amplification by PCR.

### Amplification of viral DNA genomes with replig®

In order to reduce some of the inherent biases in multi-displacement amplification (MDA), such as a preference for ssDNA viral genomes, DNA extractions were converted to dsDNA prior to amplification. Triplicate 10 μ L aliquots of the DNA extractions, containing ds and ssDNA viral genomes, underwent a single round of Klenow reaction (3′–5′ exo-, 5U/μ L) by mixing 1.5 μ L of 10× reaction buffer (New England Biolabs Buffer 2), 1.5 μ L of dNTPs (2.5 mM stock), 1 μ L of random hexamer primers (50 ng/μ L, Invitrogen). The reaction was incubated at 94°C for 3 min, then placed on ice for 3 min to allow for primer annealing before adding 1 μ L of Klenow (3′–5′ exo-) and incubated at 25°C for 10 min, then 37°C for 60 min, with a termination step of 75°C for 20 min. After termination, reactions were pooled and cleaned using a Qiagen QIAamp DNA mini kit and eluted in 50 μ L of Buffer AE. Replicate MDA reactions (*n* = 3 for each sample) were amplified using 2.5 μ L dsDNA template and the Qiagen RepliG® kit using the standard protocol. All reactions were run on a 0.8% agarose gel in 1× TAE at 100 V for 30 min to confirm amplification, pooled and cleaned with QIAampl DNA minikit and eluted in 200 μ L of Buffer AE. Negative controls were treated the same and also sent for sequencing to confirm that no viral contamination was present.

### Amplification of viral DNA genomes with RP-SISPA

As with the RepliG® protocol, Klenow Fragment (3′–5′ exo-) was used to convert all DNA genomes to dsDNA using RP-SISPA primers with a 3′ random hexamer sequence that is used for downstream PCR amplification. To label the first strand with the RP-SISPA primer, 5 μL of nucleic acid was added to 9 μL reaction mix containing 1.5 μ L of 10× PCR buffer (New England Biolabs Buffer 2); 1.0 μL of 2.5 mM dNTPs; 1.5 μL of primer FR26RV-N (GCCGGAGCTCTGCAGATATCNNNNNN, 10 μ M stock) and 5 μL of DNase-free distilled water. The reaction was incubated at 94°C for 3 min, then placed on ice for 3 min to allow for primer annealing before adding 1 μ L of Klenow Fragment (3′–5′ exo-, 5U/μ L, NEB #) and incubated at 37°C for 60 min. A second round of Klenow Fragment reaction (3′–5′ exo) labeled the second strand with the SISPA primer, by adding an additional 1 μ L of primer and 1 μ L dNTP, prior to another 94°C for 3 min heating step, then ice for 3 min before a final addition of 1 μ L of Klenow Fragment (3′–5′ exo-). The reaction was incubated at 37°C for 60 min then terminated at 75°C for 20 min.

### Amplification of viral RNA genomes with RP-SISPA

Amplification of viral RNA genomes with RP-SISPA was performed as described by Culley et al. (2010) in Manual of Aquatic Viral Ecology (MAVE). Briefly, in preparation for cDNA synthesis, 10 μ L purified RNA viral template was mixed with 1 μ L of 2.5 mM dNTPs and 1.3 μ L of FR26RV-N (GCCGGAGCTCTGCAGATATCNNNNNN, 10 μ M stock) and FR40RV-T primer (GCCGGAGCTCTGCAGATATC(T)20, 50 nM stock). The reaction was heated to 65°C for 5 min then cooled on ice for 3 min to allow the primers to anneal. While still on ice, 1 μ L DTT (Invitrogen) was added to the reaction as an enzyme stabilization reagent with 1 μ L RNase OUT (Invitrogen) to protect the sample from RNAse activity. The complementary DNA strand was synthesized with 200 U of Superscript III reverse transcriptase. The reaction was incubated initially at 25°C for 10 min to allow annealing of the hexamer 3′ end of primer FR26RV-N and the poly(T)20 3′ end of primer FR40RV-T to the template while cDNA synthesis commenced. The temperature was then increased to 50°C for 60 min. The first strand synthesis reaction was heated immediately to 94°C for 3 min and then rapidly cooled on ice. A complementary second strand was subsequently synthesized at 37°C for 60 min with the addition of 1 μ L Klenow Fragment (3′–5′ exo-, 5U/μ L). The Klenow reaction was terminated with a final incubation at 75°C for 20 min.

PCR amplification of the SISPA primer labeled template (DNA and RNA) was done in triplicate 25 μ L reactions containing 2.5 μ L 10× reaction buffer, 4 μ L dNTPs (2.5 mM stock), 2 μ L FR20RV primer (GCCGGAGCTCTGCAGATATC, 10 μ M stock), 1 μ L of template, and 0.25 μ L of TaKaRa LA HS Taq polymerase (5 U/μ L, Scientifix). The reaction was incubated at 95°C for 10 min, followed by 30 cycles of denaturation at 95°C for 30 s, 60°C for 60 s, 72°C for 90 s, and a final extension step at 72°C for 13 min to allow the completion of complementary strand synthesis. The PCR reactions were loaded on to a 0.8% agarose gel in 1×TAE at 100 V for 30 min. If amplification resulted in visible PCR products (typically a smear; products should be longer than 250 bp), a reconditioning PCR was performed on pooled reactions as follows. One reconditioning PCR contained 10 μL of pooled SISPA reaction template, 10 μL 10× buffer, 16 μL dNTP (2.5 mM stock), 8 μL FR20RV primer (10 μ M stock) and 0.75 μL TaKaRa LA HS Taq. The reaction was incubated at 95°C for 10 min, followed by 5 cycles of denaturation at 95°C for 30 s, 60°C for 60 s, 72°C for 90 s, followed by extension at 72°C for 13 min. Reactions were cleaned and QC was assessed as above.

### Sequencing and bioinformatics analysis

After amplification, samples were cleaned with a QIAamp® DNA Mini kit (RepliG® amplification) or a MinElute® PCR purification kit (RP-SISPA). Samples were checked for quantification using a Quant-iT PicoGreen® kit on a NanoDrop 3300 fluorospecrometer, for quality (260:280 ratios) on a NanoDrop 2000, and were run on a 0.8% agarose gel in 1× TAE at 100 V for 30 min to confirm a size range appropriate for sequencing (~250–500 bp) was present without contamination of smaller fragments. All metagenomes were sequenced using Nextera XT MiSeq 250 bp paired-end sequencing (Illumina) at the Ramaciotti Centre, University of New South Wales, Sydney, Australia.

Raw sequence reads were processed in CLC Genomics Workbench 5.5. Sequences were imported as Illumina paired-end reads, adaptor sequences were trimmed, and reads were checked for quality using a PHRED score of 20 and a minimum length of 100 bp. Paired reads were merged and a final data set containing merged reads and ORFans was checked again for QC with a minimum length of 200 bp. To carry out the taxonomic assignment, these non-assembled read data sets were uploaded to the Metavir web server, which is dedicated to the analysis of viral metagenomes (http://metavir-meb.univ-bpclermont.fr) (Roux et al., 2011). This server computes the taxonomic composition using tBLASTx against the NCBI viral Refseq genomes (release 2013-09-12, bit-score = 50) and normalizes the results to viral genome length using the GAAS tool (Angly et al., 2009). All virus sequences were further classified into families using the taxonomic information from the top BLAST hit. Tetranucleotide clustering and rarefaction curves were generated using tools available through Metavir. The *P. damicornis* metagenomes arose from the same biological samples and, therefore, tetranucleotide clustering was used to detect relative changes in sequence diversity caused by different methodologies. For the rarefaction curves, sequences were clustered at 75% identity because these data sets originated from the same pooled tissue homogenate sample from *P. damicornis*.

### Nucleotide sequence accession numbers

The five datasets generated from the *P. damicornis* samples were submitted to Genbank Sequence Read Archive (SRA) and are available under the accession numbers SRR1207981, SRR1207983, SRR1207980, SRR1207984, and SRR1246941(Supplementary Table 1). The two datasets generated from the *A. tenuis* samples have also been deposited in the SRA under the accession numbers SRR1207979 and SRR1210582.

## Results

### Viral DNA metagenome purification by cesium chloride density step gradient centrifugation

**Figure 2:**
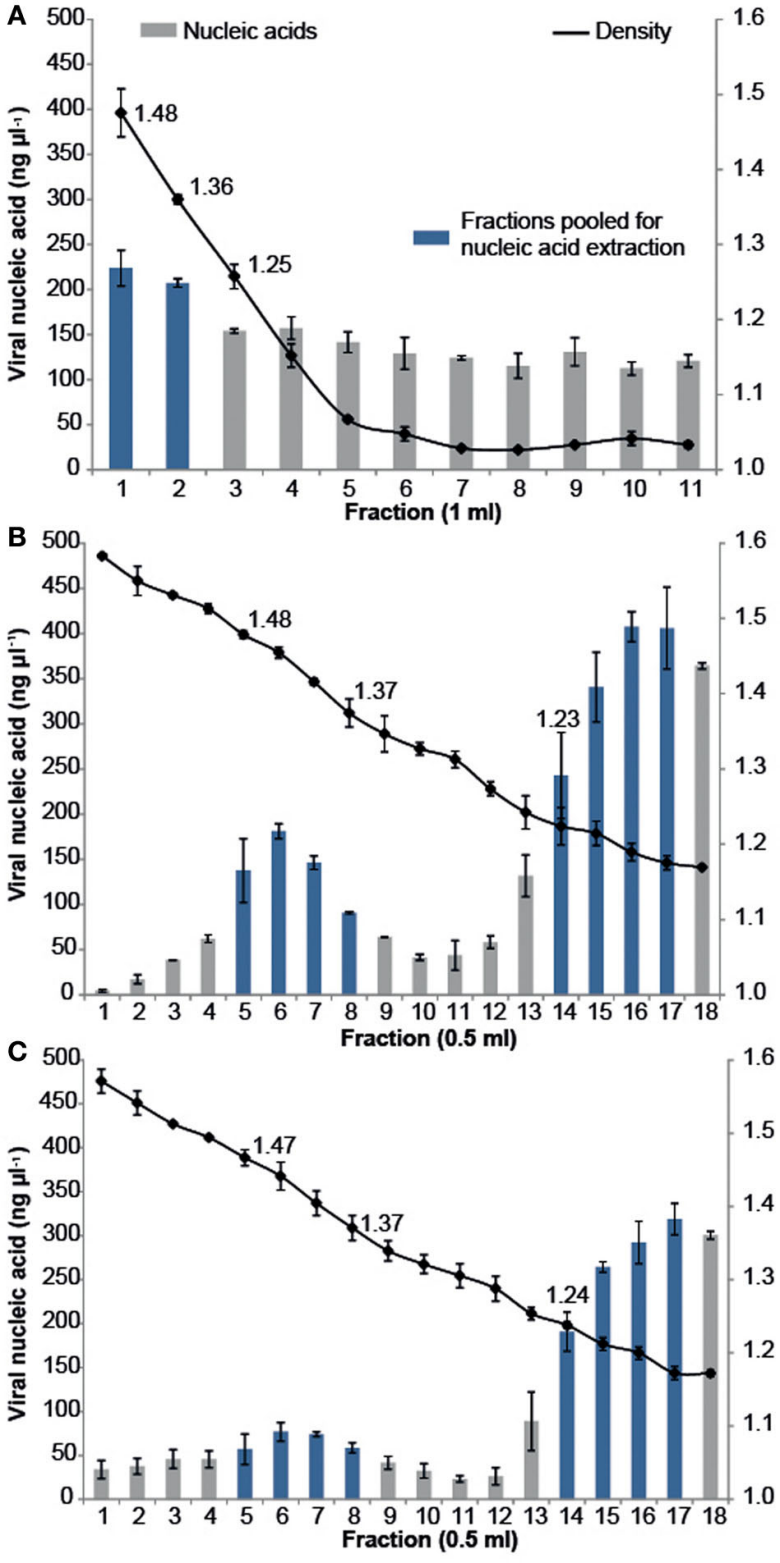
**Density and viral DNA concentrations for each fraction collected from step CsCl gradients of the virus fraction isolated from *Pocillopora damicornis* coral tissue using the (A) Chloroform (CFM) method without Liquid Nitrogen preservation of tissue (B) No Liquid Nitrogen (NLN) preservation of tissue and the MECH method (C) Liquid Nitrogen (LN2) preservation of tissue and the MECH method**. Density fractions that were pooled and used for metagenomic sequencing are highlighted in blue.

All samples were purified using cesium chloride (CsCl) density gradient centrifugation, which is a standard technique for purifying both pure virus isolates and natural virus assemblages (Lawrence and Steward, 2010). The CFM method (Marhaver et al., 2008; Thurber et al., 2008, 2009) describes purifying coral-associated viral metagenomes using a CsCl density gradient centrifugation derived from standard protocols for pure cultures of bacteriophage lambda (λ) under laboratory conditions (Sambrook et al., 1989). More recently, alternative CsCl step gradient protocols have become available that are better suited for the purification of diverse viral assemblages from environmental samples. The MECH method presented here uses a CsCl step gradient centrifugation method modified from Lawrence and Steward (2010). For all approaches, the CsCl densities and DNA quantities were measured for each sequential fraction removed along the gradient, from most to least dense (Figure 2). There was a difference in the density curve profiles generated for the CFM and MECH methods, following CsCl step-gradient centrifugation (Figure 2). The CFM method showed an initial steep decline in density in the denser half of the gradient, reaching a plateau of approximately 1.05 g mL^−1^ throughout the remaining gradient (Figure 2A). The MECH method resulted in a linear decline in density from 1.6 g mL^−1^ to a final 1.17 g mL^−1^ (Figures 2B,C).

Nucleic-acid quantification by Pico Green assay showed the CFM method had an even distribution of nucleic acids (fractions yielded between 120 and 220 ng μl^−1^ DNA) throughout the gradient, while the MECH method resulted in a bimodal distribution with peaks at ~1.4 g mL^−1^ and ~1.2 g mL^−1^ for both LN2 (80–320 ng μl^−1^ DNA) and NLN treatments (~200–400 ng μl^−1^ DNA) (Figures 2B,C). The published CFM method (Marhaver et al., 2008; Thurber et al., 2008, 2009) did not include gravimetric measurements of density of the collected gradient fractions, but stated that a 1.5 mL fraction should be removed from just below the original 1.5 g mL^−1^ step marked on the outside of the tube during layering (Thurber et al., 2009). As a literal interpretation of the protocol, we measured the density of each fraction in the CFM CsCl step gradient and pooled the recommended density range of 1.35–1.5 g mL^−1^ (fractions 1, 2) (Figure 2A) before proceeding to viral nucleic-acid extraction and sequencing. It has been reported that the 1.35–1.5 g mL^−1^ fraction contains bacteriophages (King et al., 2011), but this fraction alone will not recover a wide diversity of the viruses present (Breitbart et al., 2002). Nucleic acid quantification of each fraction assists in deciding which fractions to target and pool for viral nucleic-acid extraction and sequencing as was done for both NLN and LN2 samples. For example, we pooled fractions 5–8 and 14–17 (Figure 2B) for the NLN samples, which corresponds to buoyant densities of bacteriophages and viruses that infect eukaryotes, respectively (King et al., 2011). Although the pattern of CsCl step gradient profiles does not change, we found the DNA yield was 25–50% lower as a consequence of LN2 treatment of coral tissues compared to the NLN treatment (Figure 2C).

In the MECH method, following removal and pooling of the indicated fractions from each CsCl step gradient, diafiltration was performed to remove CsCl salts. Diafiltration, involving several buffer exchange steps with 0.02 μm filtered SM buffer to remove CsCl salts following density gradient centrifugation, has not previously been reported for coral viral metagenome isolation, although it is used in other aquatic virus isolation techniques. Following diafiltration, the pooled fractions were 0.2 μm Durapore® filtered to remove any residual cellular material. Although this may exclude some large viruses, the Durapore® membrane type allows smaller viruses to pass through and ensures residual cellular contamination is removed, a problem best dealt with prior to sequencing (Roux et al., 2013). Analysis of the metagenomic data sets using a HMM (Hidden Markov Model)-based detection tool revealed that 16S rDNA sequences (proxy for detection of cellular DNA contamination in viral metagenomes) (Roux et al., 2013) were less than 2 in 10,000 sequences (Supplementary Table 1).

### Coral-associated viral metagenomic diversity

**Figure 3:**
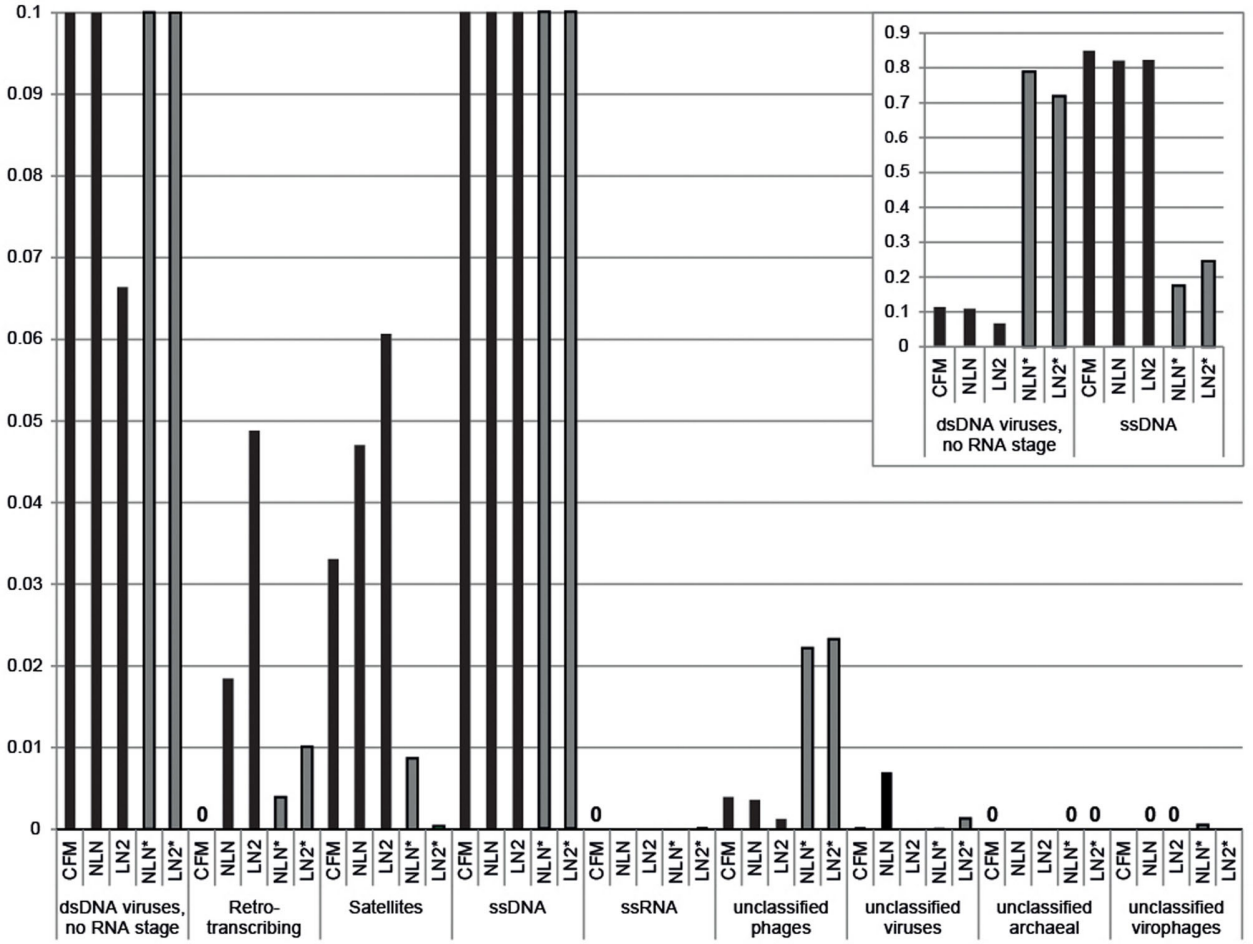
**Annotation of *Pocillopora damicornis* DNA virome metagenomic sequences with a match to viral sequences for each method and treatment**. Best matches to major viral groups were examined through Metavir, which uses tBLASTx algorithm against the NCBI viral Refseq genomes with a bit-score of 50 (data sets are publically available at http://metavir-meb.univ-bpclermont.fr, project “Coral virus—generating metagenomes”). Total numbers (per cent) of sequences for each viral group are indicated in Table 1. All samples were amplified using a RepliG® kit except for samples NLN^*^ and LN2^*^, which underwent RP-SISPA amplification.

Viral DNA was extracted from *P. damicornis* colonies (*n* = 3), pooled and prepared for sequencing according to the CFM or MECH protocol. The nucleic acids in the viral fraction purified from coral tissues are typically not in high enough concentration for sequencing, and therefore need to be amplified prior to sequencing. We tested a combination of different amplification methods, as described, and compared the resulting metagenomic data. Information on the reads generated from the Illumina MiSeq sequencing is shown in Supplementary Table 1. Following sequencing, the proportion of sequences that matched to known viral sequences in the Genbank non-redundant database using tBLASTx (bit-score = 50) is shown in Table 1 and Figure 3. RP-SISPA-amplified samples yielded a higher total proportion of dsDNA viral-like sequence hits compared to amplification using RepliG® (Table 1). The largest proportion of sequence similarities in the dsDNA virus group was to bacteriophages (93.5–98.6% for RepliG®-amplified samples and ~99% for RP-SISPA-amplified samples) (Figure 3; Table 1). Amplification of samples using RepliG® resulted in a high proportion of hits to ssDNA viruses (Table 1), which is typical for MDA-amplified viral DNA from aquatic environments (Kim and Bae, 2011). However, relative to the CFM method, the RepliG®-amplified MECH samples had a higher proportion of sequences with similarity to dsDNA eukaryotic viruses. Notably, the CFM metagenomes exhibited the lowest diversity, with no identified representatives for retro-transcribed (RT) and ssRNA viruses (Figure 3; Table 1). For both amplification techniques, the LN2 metagenomes had the highest proportion of hits to RT viruses (Table 1; Figure 3). Overall, ssRNA and unclassified archaeal viruses were absent from most samples, with only a very low percentage of hits seen in the NLN and LN2 MECH metagenomes (Table 1).

**Table 1:** **Percentage of viruses identified in DNA viromes by BLAST comparison to the NCBI viral Refseq genome database (bit-score = 50, GAAS normalized)**.

**Virus type**	**CFM**	**MECH**			
		**NLN**	**LN2**	NLN*	LN2*
dsDNA viruses, no RNA stage	11.3	10.9	6.6	78.9	71.9
Bacteriophage	(98.6%)	(93.5%)	(93.5%)	(99%)	(99%)
Eukaryotic	(1.4%)	(6.5%)	(6.5%)	(1%)	(1%)
Reverse transcribing viruses	0	1.8	4.9	0.4	1.0
Satellites	3.3	4.7	6.1	0.9	0.1
ssDNA viruses	84.9	82.1	82.3	17.5	24.6
ssRNA viruses	0	<0.1	<0.1	<0.1	<0.1
Unclassified archaeal viruses	0	<0.1	0	0	<0.1
Unclassified phages	0.4	0.4	0.1	2.2	2.3
Unclassified viruses	<0.1	0.1	<0.1	<0.1	0.1
Virophage	<0.1	0	0	<0.1	<0.1

The viral metagenomic data sets generated by the previously published chloroform treatment method (CFM) and our novel mechanical disruption method (MECH). The latter method was tested with (LN2) and without (NLN) liquid nitrogen tissue preservation. Data sets with an asterisk (^*^) were amplified using RP-SISPA prior to sequencing and were analyzed after removal of contaminating ssDNA phiX sequences (spiked during Illumina sequencing). All other data sets were amplified using RepliG® technology. Percent of dsDNA viruses as phage or eukaryotic viruses are highlighted in brackets.

**Figure 4:**
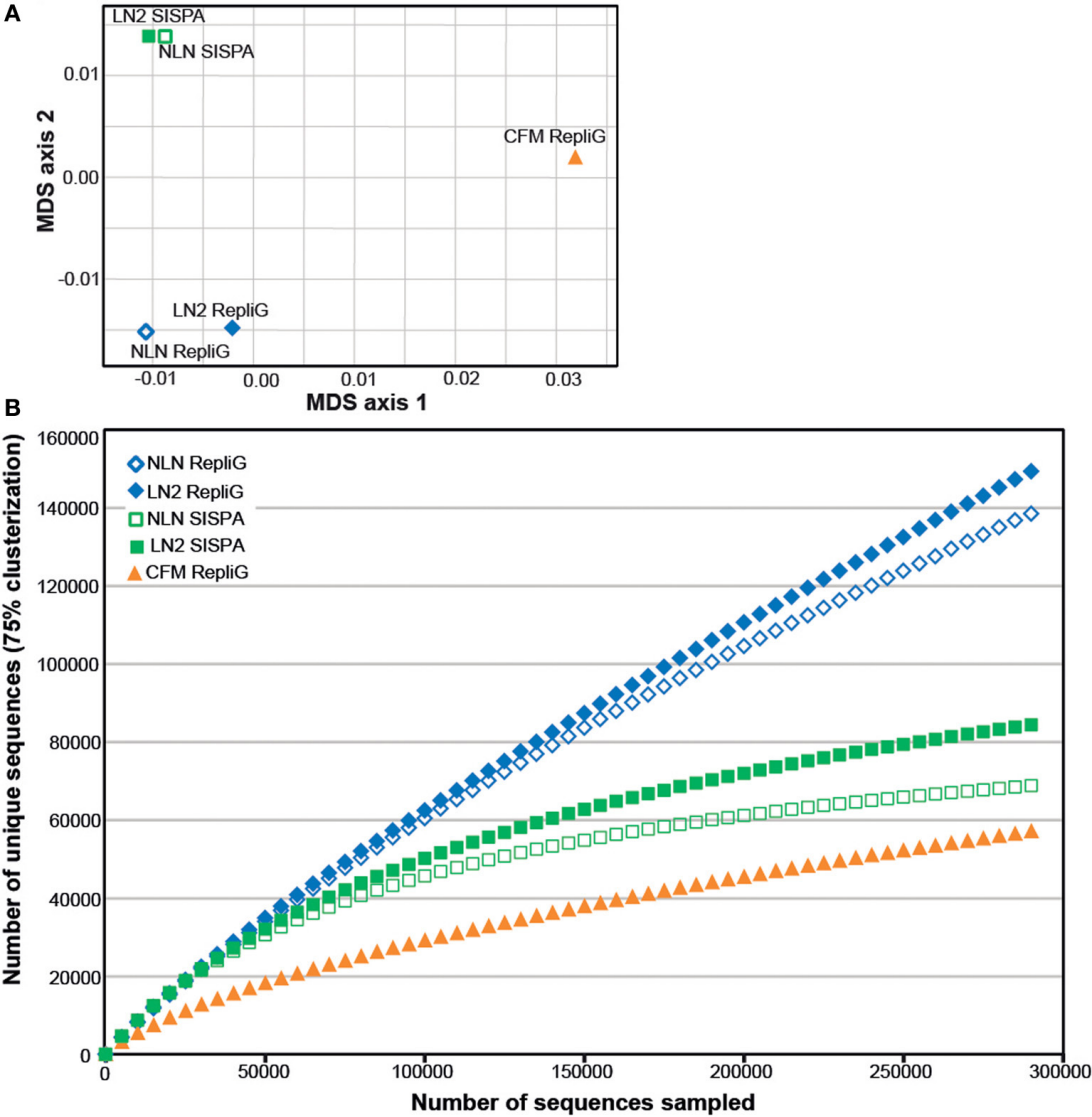
**Diversity analysis of the *Pocillopora damicornis* DNA virome data sets**. Each data set was generated from a uniform tissue homogenate using either a chloroform-based method (CFM) or a mechanical bead-beating method (MECH) to disrupt cells. The latter method included either an additional liquid nitrogen storage step (LN2) or samples were processed immediately from fresh tissue (NLN). The nucleic acid amplification method used for each data set is indicated (RepliG® or RP-SISPA amplification). **(A)** tetranucleotide cluster analysis was used to demonstrate how the methods change nucleotide frequency (proxy for viral community diversity) **(B)** rarefaction analysis was used to illustrate the impact of methodology on perceived diversity within a viral metagenome.

As an alternative way of assessing viral assemblage similarities and differences, we conducted tetranucleotide clustering on the viral sequences identified from the metagenomes (Figure 4A). Since the metagenomes originated from the same pooled coral tissue samples and therefore should have a similar oligonucleotide signature, we used tetranucleotides as a more sensitive approach than dinucleotides, as demonstrated previously (Pride et al., 2003; Teeling et al., 2004). This analysis shows that both the method of cell disruption during virus isolation, as well as the nucleic-acid amplification method prior to sequencing, affects the viral metagenome composition. The NLN and LN2 MECH metagenomes clustered based on RepliG® or RP-SISPA amplification, but metagenomes from the CFM method (also amplified with RepliG®) had a tetranucleotide signal that was distinct from all other data sets (Figure 4A). To estimate the fraction of virus types sequenced and compare viral sequence diversity per unit sampling effort, we generated rarefaction curves (Figure 4B). Rarefaction curves plot the sequence clusters randomly sampled from a metagenomic data set as a function of the total number of sequence clusters sampled. The rarefaction curves sequences clustered at 75% similarity (Roux et al., 2011) showed that the CFM method had lower sequence diversity overall (Figure 4B), especially when compared to the MECH RepliG® amplified samples (Figure 3). The MECH samples amplified by RepliG® had higher sequence diversity than those amplified with RP-SISPA, with rarefaction curves not saturating after 300,000 randomly sampled sequences. These results indicate that biases in coral viral metagenomes are caused by the genome amplification method, as well as other steps in viral nucleic acid isolation.

**Figure 5:**
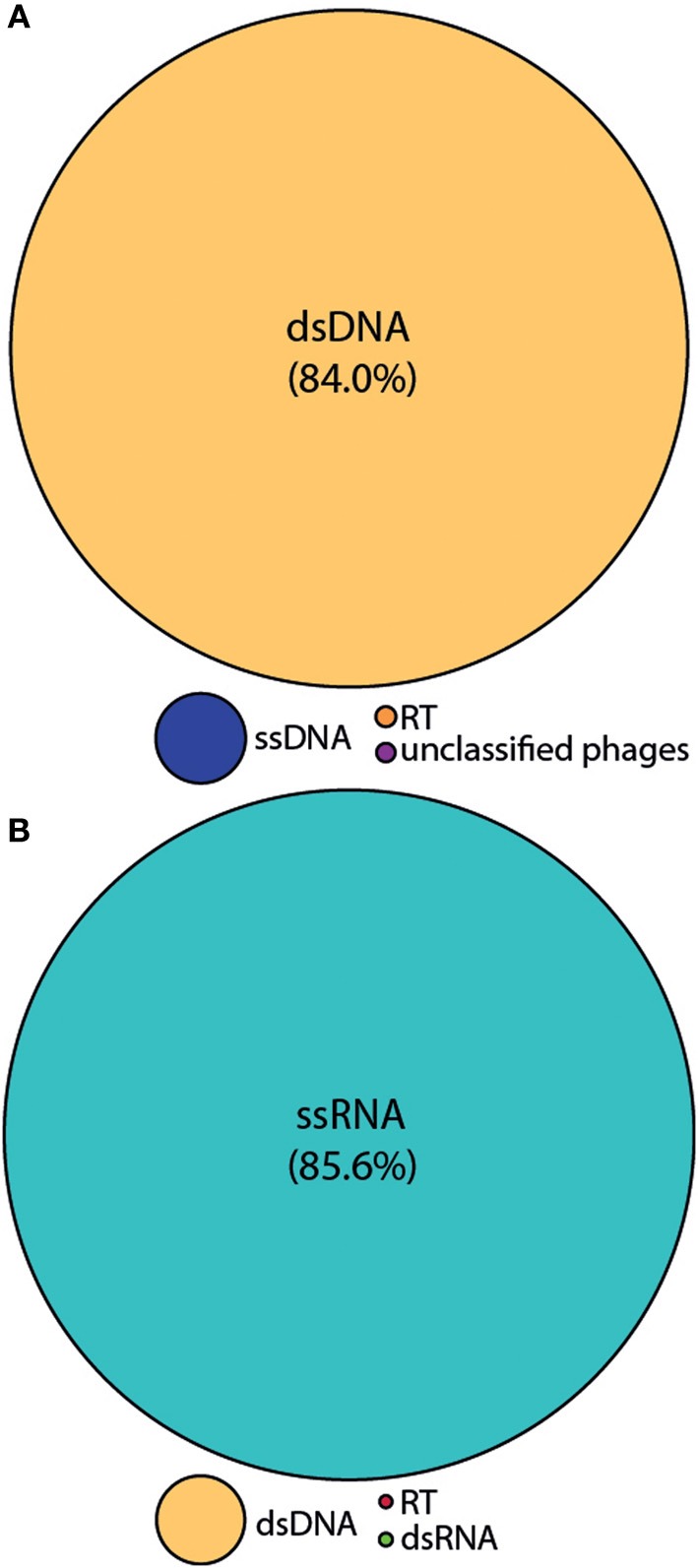
**Annotation of the *Acropora tenuis* (A) DNA and (B) RNA virome metagenomic sequences with a match to viral sequences for the MECH method following storage in liquid nitrogen and amplified by RP-SISPA**. Best matches to the major viral groups were examined through Metavir, which uses the tBLASTx algorithm against the NCBI viral Refseq genomes with a bit-score of 50.

We employed the MECH method for both DNA and RNA viromes in five other coral species and present viral diversity data here for one of these, *Acropora tenuis*. The samples were collected at a remote field location and tissue homogenates were stored in liquid nitrogen prior to MECH. We amplified the DNA and RNA-virus fractions using the modified RP-SISPA method described here and an existing RNA RP–SISPA protocol (Culley et al., 2010), respectively. The diversity data for DNA viruses generated using our method, revealed that 84% of the sequences with hits were for dsDNA viruses (Figure 5A). The RNA viral sequences generated from *A. tenuis* were ~85.6% from ssRNA viruses (Figure 5B), with the majority of the identified hits (~99%) matching a major capsid protein (MCP) gene from the dinoflagellate-infecting ssRNA virus, *Heterocapsa circularisquama* RNA virus (HcRNAV) (bit-score = 50). This result supports the observation of sequence reads with similarities to HcRNAV previously reported in a *Montastrea cavernosa* study (Correa et al., 2013).

## Discussion

We have developed and optimized a method for the extraction and preparation of viral DNA and RNA from coral tissues for metagenome sequencing. Metagenomics can be a powerful tool for describing biological diversity in a sample without prior information on the nature of the sample. However, the biological relevance and accuracy of metagenomic sequence data can be reflective of how nucleic acids (i.e., the metagenome) were obtained. We show that the methods used in viral nucleic acids preparation can introduce severe biases. The method presented here minimizes biases and captures a larger fraction of the viral diversity in coral samples compared to previously published methods.

Previously described methods for isolating viral nucleic acids from coral tissue have included a chloroform step in order to disrupt cellular material (Marhaver et al., 2008; Thurber et al., 2008; Hewson et al., 2012). Certain virus types are sensitive to chloroform and other organic solvents, which denature the lipid envelopes surrounding virus capsids (Feldman and Wang, 1961; Ackermann, 2006), and internal lipid membranes (Bamford et al., 1995), which are also a common feature of phycoviruses (Dunigan et al., 2006; Wilson et al., 2009) and other nucleo-cytoplasmic large DNA (NCLDV) viruses. Furthermore, viruses that do not possess a lipid envelope, such as ssDNA viruses belonging to the *Inoviridae*, and about a third of tailed bacteriophages (Ackermann, 2006) also display sensitivity to chloroform. Disruption of the lipid envelope surrounding a virus is typically detected by a decrease in infectivity, but it also changes the physical attributes of the virus including its size and buoyancy. Changes in the physical characteristics of a virus will directly affect outcomes of isolation and can lead to the exclusion of certain members of the viral community from sample preparations. We postulated that in order to capture as many representative virus groups as possible, chloroform should be avoided. To test this, a previously reported method (Marhaver et al., 2008; Thurber et al., 2008, 2009) incorporating a chloroform addition step (CFM) was compared to the mechanical homogenization bead-beating (MECH) approach described here.

Notably, there is a loss of sequence data for reverse-transcribing viruses in the chloroform-treated samples, likely because reverse-transcribing viruses have a lipid envelope. This is also a common feature of viruses in several other families (e.g., *Herpesviridae, Poxviridae*) (King et al., 2011). Therefore, chloroform addition during the purification of viruses from coral tissue will affect the types of viruses present in the metagenome and results in lower diversity compared to the samples treated with the MECH approach. In addition, the CsCl step-gradient centrifugation described in our method maximizes the capture of a wider diversity of viruses because it includes density and nucleic acid quantification for each fraction removed along the gradient to ensure that the appropriate fractions are targeted.

Full processing of coral tissue after collection is often delayed, necessitating the use of liquid nitrogen for preservation. Our results demonstrate that storage of coral-tissue homogenate in liquid nitrogen prior to purifying viruses and extracting nucleic acids can result in lower starting yields of nucleic acids but does not significantly alter the representative sequences in the metagenomic data.

Amplification methods cause artifacts and biases. We recommend the use of the RP-SISPA methods presented here for amplifying viral DNA and RNA isolated from coral tissue using mechanical disruption. RP-SISPA has been used previously to detect and identify viruses through a metagenomic approach (Culley et al., 2010; Karlsson et al., 2013), and has an advantage over MDA in that branching does not occur during amplification, the formation of chimeras is reduced and ssDNA is not preferentially amplified (Polson et al., 2011). However, RP-SISPA is not without its own issues and has been reported to show a template-dependent amplification bias, resulting in uneven sequencing depth within and among genomes (Victoria et al., 2009, 2010; Rosseel et al., 2013). RP-SISPA can have a bias for amplifying the dominant sequences in metagenomic samples (Karlsson et al., 2013), likely the reason that most of the sequences in the RP-SISPA metagenomes contain sequences from the dsDNA viruses, which have larger genomes relative to other viral types. In addition, PCR amplification is known to produce amplification artifacts, such as increased heteroduplex formation in samples with increased diversity (Qiu et al., 2001). Because of this, we have included a reconditioning PCR approach, where replicate amplification PCR reactions are pooled and then “reconditioned” through a low number of additional cycles in order to minimize heteroduplex formation (Thompson et al., 2002) and eliminate potential amplification biases within a single PCR reaction. Although our data indicate that RP-SISPA is preferred over MDA, caution must be exercised in order to avoid over-interpreting metagenomic data generated following amplification, especially regarding relative abundances of viral groups.

The poor representation of many viral taxa in public databases limits the ability to interpret the results from metagenomic sequence data. For example, low levels of archaeal viruses in our coral viromes may result from the lack of representative sequences in the public databases. In all samples, the majority of dsDNA hits were to bacteriophages, which mirrors the findings in other metagenomic studies of viruses in reef corals (Marhaver et al., 2008; Thurber et al., 2008). It is notable that in the RNA metagenome data for *A. tenuis* there was a high proportion of hits matching a MCP gene found in a ssRNA algal virus (HcRNAV) that infects dinoflagellates (Tomaru et al., 2004). This indicates the potential presence of a virus associated with the dinoflagellate endosymbiont, *Symbiodinium*, in the coral holobiont and supports the observation of sequence reads with similarities to HcRNAV reported in a previous study of coral-associated viruses (Correa et al., 2013). As viral genomes can be DNA or RNA, double- or single-stranded (King et al., 2011), coral viromic studies should try to extract both RNA and DNA viruses in an attempt to capture as much of the virus community as possible.

## Conclusion

We developed a method to generate viral metagenomes from the coral holobiont. This new method uses a mechanical approach to disrupt host cells and avoids the use of chemicals, such as chloroform, which we demonstrate can reduce the quantity and diversity of the viruses targeted. We also note that caution must be exercised when drawing inferences from viral metagenome data generated using currently available amplification methods, due to possible introduced biases in the processing prior to sequencing. The method presented here has been tested on coral species from different taxonomic families and performs well in uncovering a wider diversity of the viromes present in coral tissue samples.

### Conflict of interest statement

The authors declare that the research was conducted in the absence of any commercial or financial relationships that could be construed as a potential conflict of interest.

## References

[B1] Ackermann H. W. (2006). Classification of bacteriophages, in The Bacteriophages, 2nd Edn ed Calendar R. (New York, NY: Oxford University Press), 8–16

[B2] Angly F. E. Felts B. Breitbart M. Salamon P. Edwards R. A. Carlson C. (2006). The marine viromes of four oceanic regions. PLoS Biol. 4:e368 10.1371/journal.pbio.0040368 17090214 PMC1634881

[B3] Angly F. E. Willner D. Prieto-Davo A. Edwards R. A. Schmieder R. Vega-Thurber R. (2009). The GAAS metagenomic tool and its estimations of viral and microbial average genome size in four major biomes. PLoS Comput. Biol. 5:e1000593 10.1371/journal.pcbi.1000593 20011103 PMC2781106

[B4] Bamford D. H. Caldentey J. Bamford J. K. H. (1995). Bacteriophage PRD1 - a broad-host-range dsDNA tectivirus with an internal membrane. Adv. Virus Res. 45, 281–319 10.1016/s0065-3527(08)60064-0 7793328

[B5] Breitbart M. Salamon P. Andresen B. Mahaffy J. M. Segall A. M. Mead D. (2002). Genomic analysis of uncultured marine viral communities. Proc. Natl. Acad. Sci. U.S.A. 99, 14250–14255 10.1073/pnas.202488399 12384570 PMC137870

[B6] Correa A. M. S. Welsh R. M. Thurber R. L. V. (2013). Unique nucleocytoplasmic dsDNA and +ssRNA viruses are associated with the dinoflagellate endosymbionts of corals. ISME J. 7, 13–27 10.1038/ismej.2012.75 22791238 PMC3526182

[B7] Culley A. I. Suttle C. A. Steward G. F. (2010). Characterization of the diversity of marine RNA viruses, in Manual of Aquatic Viral Ecology, eds Wilhelm S. W. Weinbauer M. G. Suttle C. A. (Waco, TX: ASLO), 193–201

[B8] Davy J. E. Patten N. L. (2007). Morphological diversity of virus-like particles within the surface microlayer of scleractinian corals. Aquat. Microb. Ecol. 47, 37–44 10.3354/ame047037

[B9] Davy S. K. Burchett S. G. Dale A. L. Davies P. Davy J. E. Muncke C. (2006). Viruses: agents of coral disease? Dis. Aquat. Org. 69, 101–110 10.3354/dao069101 16703772

[B10] Dinsdale E. A. Pantos O. Smriga S. Edwards R. A. Angly F. Wegley L. (2008). Microbial ecology of four coral atolls in the northern line islands. PLoS ONE 3:e1584 10.1371/journal.pone.0001584 18301735 PMC2253183

[B11] Dunigan D. D. Fitzgerald L. A. Van Etten J. L. (2006). Phycodnaviruses: a peek at genetic diversity. Virus Res. 117, 119–132 10.1016/j.virusres.2006.01.024 16516998

[B12] Efrony R. Atad I. Rosenberg E. (2009). Phage therapy of coral white plague disease: properties of phage BA3. Curr. Microbiol. 58, 139–145 10.1007/s00284-008-9290-x 18923867

[B13] Efrony R. Loya Y. Bacharach E. Rosenberg E. (2007). Phage therapy of coral disease. Coral Reefs 26, 7–13 10.1007/s00338-006-0170-1

[B14] Feldman H. A. Wang S. S. (1961). Sensitivity of various viruses to chloroform. Proc. Soc. Exp. Biol. Med. 106, 736–738 13698695 10.3181/00379727-106-26459

[B15] Hewson I. Brown J. M. Burge C. A. Couch C. S. Labarre B. A. Mouchka M. E. (2012). Description of viral assemblages associated with the *Gorgonia ventalina* holobiont. Coral Reefs 31, 487–491 10.1007/s00338-011-0864-x PMC7087884 32214633

[B16] Karlsson O. E. Belak S. Granberg F. (2013). The effect of preprocessing by Sequence-Independent Single-Primer Amplification (SISPA) on metagenomic detection of viruses. Biosecur. Bioterror. 11, S227–S234 10.1089/bsp.2013.0008 23971810

[B17] Kim K. H. Bae J. W. (2011). Amplification methods bias metagenomic libraries of uncultured single-stranded and double-stranded DNA viruses. Appl. Environ. Microbiol. 77, 7663–7668 10.1128/aem.00289-11 21926223 PMC3209148

[B18] King A. M. Q. Adams M. J. Carstens E. B. Lefkowitz E. J. (2011). Virus Taxonomy: Ninth Report of the International Committee on Taxonomy of Viruses. San Diego, CA: Elsevier Academic Press

[B19] Lawrence J. E. Steward G. F. (2010). Purification of viruses by centrifugation, in Manual of Aquatic Viral Ecology, eds Wilhelm S. W. Weinbauer M. G. Suttle C. A. (Waco, TX: ASLO), 166–181

[B20] Lohr J. Munn C. B. Wilson W. H. (2007). Characterization of a latent virus-like infection of symbiotic zooxanthellae. Appl. Environ. Microbiol. 73, 2976–2981 10.1128/aem.02449-06 17351090 PMC1892877

[B21] Marhaver K. L. Edwards R. A. Rohwer F. (2008). Viral communities associated with healthy and bleaching corals. Environ. Microbiol. 10, 2277–2286 10.1111/j.1462-2920.2008.01652.x 18479440 PMC2702503

[B22] Patten N. L. Harrison P. L. Mitchell J. G. (2008a). Prevalence of virus-like particles within a staghorn scleractinian coral (*Acropora muricata*) from the Great Barrier Reef. Coral Reefs 27, 569–580 10.1007/s00338-008-0356-9

[B23] Patten N. L. Mitchell J. G. Middelboe M. Eyre B. D. Seuront L. Harrison P. L. (2008b). Bacterial and viral dynamics during a mass coral spawning period on the Great Barrier Reef. Aquat. Microb. Ecol. 50, 209–220 10.3354/ame01179

[B24] Patten N. L. Seymour J. R. Mitchell J. G. (2006). Flow cytometric analysis of virus-like particles and heterotrophic bacteria within coral-associated reef water. J. Mar. Biol. Assoc. U.K. 86, 563–566 10.1017/s0025315406013476

[B25] Polson S. W. Wilhelm S. W. Wommack K. E. (2011). Unraveling the viral tapestry (from inside the capsid out). ISME J. 5, 165–168 10.1038/ismej.2010.81 20555364 PMC3105704

[B26] Pride D. T. Meinersmann R. J. Wassenaar T. M. Blaser M. J. (2003). Evolutionary implications of microbial genome tetranucleotide frequency biases. Genome Res. 13, 145–158 10.1101/gr.335003 12566393 PMC420360

[B27] Qiu X. Y. Wu L. Y. Huang H. S. McDonel P. E. Palumbo A. V. Tiedje J. M. (2001). Evaluation of PCR-generated chimeras: mutations, and heteroduplexes with 16S rRNA gene-based cloning. Appl. Environ. Microbiol. 67, 880–887 10.1128/aem.67.2.880-887.2001 11157258 PMC92662

[B28] Rohwer F. Prangishvili D. Lindell D. (2009). Roles of viruses in the environment. Environ. Microbiol. 11, 2771–2774 10.1111/j.1462-2920.2009.02101.x 19878268

[B29] Rohwer F. Seguritan V. Azam F. Knowlton N. (2002). Diversity and distribution of coral- associated bacteria. Mar. Ecol. Prog. Ser. 243, 1–10 10.3354/meps243001

[B30] Rosseel T. Van Borm S. Vandenbussche F. Hoffmann B. Van Den Berg T. Beer M. (2013). The origin of biased sequence depth in sequence-independent nucleic acid amplification and optimization for efficient massive parallel sequencing. PLoS ONE 8:e76144 10.1371/journal.pone.0076144 24086702 PMC3784409

[B31] Roux S. Faubladier M. Mahul A. Paulhe N. Bernard A. Debroas D. (2011). Metavir: a web server dedicated to virome analysis. Bioinformatics 27, 3074–3075 10.1093/bioinformatics/btr519 21911332

[B32] Roux S. Krupovic M. Debroas D. Forterre P. Enault F. (2013). Assessment of viral community functional potential from viral metagenomes may be hampered by contamination with cellular sequences. Open Biol. 3, 1–13 10.1098/rsob.130160 24335607 PMC3877843

[B33] Sambrook J. Fritsch E. F. Maniatis T. (1989). Molecular Cloning. A Laboratory Manual. New York, NY: Cold Spring Harbor Laboratory Press

[B34] Seymour J. R. Patten N. Bourne D. G. Mitchell J. G. (2005). Spatial dynamics of virus-like particles and heterotrophic bacteria within a shallow coral reef system. Mar. Ecol. Prog. Ser. 288, 1–8 10.3354/meps288001

[B35] Suttle C. A. (2005). Viruses in the sea. Nature 437, 356–361 10.1038/nature04160 16163346

[B36] Suttle C. A. (2007). Marine viruses - major players in the global ecosystem. Nat. Rev. Microbiol. 5, 801–812 10.1038/nrmicro1750 17853907

[B37] Teeling H. Meyerdierks A. Bauer M. Amann R. Glockner F. O. (2004). Application of tetranucleotide frequencies for the assignment of genomic fragments. Environ. Microbiol. 6, 938–947 10.1111/j.1462-2920.2004.00624.x 15305919

[B38] Thompson J. R. Marcelino L. A. Polz M. F. (2002). Heteroduplexes in mixed-template amplifications: formation, consequence and elimination by ‘reconditioning PCR.’ Nucleic Acids Res. 30, 2083–2088 10.1093/nar/30.9.2083 11972349 PMC113844

[B39] Thurber R. L. V. Barott K. L. Hall D. Liu H. Rodriguez-Mueller B. Desnues C. (2008). Metagenomic analysis indicates that stressors induce production of herpes-like viruses in the coral *Porites compressa*. Proc. Natl. Acad. Sci. U.S.A. 105, 18413–18418 10.1073/pnas.0808985105 19017800 PMC2584576

[B40] Thurber R. L. V. Haynes M. Breitbart M. Wegley L. Rohwer F. (2009). Laboratory procedures to generate viral metagenomes. Nat. Protoc. 4, 470–483 10.1038/nprot.2009.10 19300441

[B41] Tomaru Y. Katanozaka N. Nishida K. Shirai Y. Tarutani K. Yamaguchi M. (2004). Isolation and characterization of two distinct types of HcRNAV, a single-stranded RNA virus infecting the bivalve-killing microalga *Heterocapsa circularisquama*. Aquat. Microb. Ecol. 34, 207–218 10.3354/ame034207

[B42] Veron J. E. N. (1993). Corals of Australia and the Indo-Pacific. Honolulu, HI: University of Hawai'i Press

[B43] Victoria J. G. Kapoor A. Li L. L. Blinkova O. Slikas B. Wang C. L. (2009). Metagenomic analyses of viruses in stool samples from children with acute flaccid paralysis. J. Virol. 83, 4642–4651 10.1128/jvi.02301-08 19211756 PMC2668503

[B44] Victoria J. G. Wang C. L. Jones M. S. Jaing C. McLoughlin K. Gardner S. (2010). Viral nucleic acids in live-attenuated vaccines: detection of minority variants and an adventitious virus. J. Virol. 84, 6033–6040 10.1128/jvi.02690-09 20375174 PMC2876658

[B45] Weynberg K. D. Allen M. J. Gilg I. C. Scanlan D. J. Wilson W. H. (2011). Genome sequence of *Ostreococcus tauri* virus OtV-2 throws light on the role of picoeukaryote niche separation in the ocean. J. Virol. 85, 4520–4529 10.1128/jvi.02131-10 21289127 PMC3126241

[B46] Wilson W. H. Dale A. L. Davy J. E. Davy S. K. (2005a). An enemy within? Observations of virus-like particles in reef corals. Coral Reefs 24, 145–148 10.1007/s00338-004-0448-0

[B47] Wilson W. H. Francis I. Ryan K. Davy S. K. (2001). Temperature induction of viruses in symbiotic dinoflagellates. Aquat. Microb. Ecol. 25, 99–102 10.3354/ame025099

[B48] Wilson W. H. Schroeder D. C. Allen M. J. Holden M. T. G. Parkhill J. Barrell B. G. (2005b). Complete genome sequence and lytic phase transcription profile of a coccolithovirus. Science 309, 1090–1092 10.2307/3842556 16099989

[B49] Wilson W. H. Van Etten J. L. Allen M. J. (2009). The *Phycodnaviridae*: the story of how tiny giants rule the world, in Lesser Known Large dsDNA Viruses, ed Van Etten J. L. (Berlin; Heidelberg: Springer-Verlag), 1–42 10.1007/978-3-540-68618-7_1 PMC2908299 19216434

[B50] Wommack K. E. Colwell R. R. (2000). Virioplankton: viruses in aquatic ecosystems. Microbiol. Mol. Biol. Rev. 64, 69 10.1128/mmbr.64.1.69-114.2000 10704475 PMC98987

