# Supplementary material for: Generating viral metagenomes from the coral holobiont
Source: Front Microbiol. 2014 May 7;5:206. doi: 10.3389/fmicb.2014.00206 (PMC4019844; doi:10.3389/fmicb.2014.00206)
Supplement: Supplementary file 1 [file DataSheet1.DOCX]

**Supplementary Table 1:** Summary of sequence data sets produced using different methods for generating viral metagenomes from coral tissue (species used here was *Pocillopora damicornis*). The ratio of rDNA genes was determined to be below 2 in 10,000 sequences ([Roux et al., 2013](#_ENREF_32)).

| **Sample Preparation** | **Amplification** | **Illumina 250bp paired-end reads, QCº** | **NBCI accession number** | **Merged, QCº, trim to 200bp** | **Subsampled for Metavir analysis*^** | **16S** | **Per cent 16S** |
| --- | --- | --- | --- | --- | --- | --- | --- |
| Chloroform treated (CFM) | MDA | 1,438,584 | SRR1207980 | 370,153 | 370,153 | 30 | 0.0081% |
| Fresh blastate (NLN) | MDA | 1,211,150 | SRR1207981 | 435,494 | 435,494 | 133 | 0.0305% |
|  | RP-SISPA | 7,908,406 | SRR1207984 | 2,965,665 | 2,000,000 | 9 | 0.0005% |
| Frozen blastate (LN2) | MDA | 898,894 | SRR1207983 | 292,476 | 292,476 | 85 | 0.0291% |
|  | RP-SISPA | 8,638,016 | SRR1246941 | 2,646,987 | 2,000,000 | 147 | 0.0074% |

ºQC PHRED = 20, max 2 ambiguous nucleotides, trim 3 nucleotides from 3' end, removal of MiSeq adaptors/SISPA primers (where appropriate), trim to 100bp

*Available at Metavir (http://metavir-meb.univbpclermont.fr) under Coral virus – generating metagenomes project; also available at Genbank Sequence Read Archive (accession numbers as shown)

^2 million was data set limit for Metavir at the time of analysis
